# Innovation of BiOBr/BiOI@Bi_5_O_7_I Ternary Heterojunction for Catalytic Degradation of Sodium P-Perfluorous Nonenoxybenzenesulfonate

**DOI:** 10.3390/toxics12040298

**Published:** 2024-04-17

**Authors:** Tao Xu, Yang Liu, Tie-Qing You, Jia Bao

**Affiliations:** School of Environmental and Chemical Engineering, Shenyang University of Technology, Shenyang 110870, China

**Keywords:** photocatalysis, ternary heterojunction, OBS, catalyst, degradation

## Abstract

As an alternative for perfluorooctane sulfonic acid (PFOS), sodium p-perfluorononyloxybenzene sulfonate (OBS) has been widely used in petroleum, fire-fighting materials, and other industries. In order to efficiently and economically remove OBS contaminations from water bodies, in this study, a ternary heterojunction was constructed by coupling BiOBr and BiOI@Bi_5_O_7_I for improving the redox capacity and carrier separation ability of the material and investigating the effect of the doping ratios of BiOBr and BiOI@ Bi_5_O_7_I on the performance of the catalysts. Furthermore, the effects on the degradation of OBS were also explored by adjusting different catalyst doping ratios, OBS concentrations, catalyst amounts, and pH values. It was observed that when the concentration of OBS was 50 mg/L, the amount of catalyst used was 0.5 g/L, and the pH was not changed. The application of BiOBr/BiOI@ Bi_5_O_7_I consisting of 25% BiOBr and 75% BiOI@ Bi_5_O_7_I showed excellent stability and adsorption degradation performance for OBS, and almost all of the OBS in the aqueous solution could be removed. The removal rate of OBS by BiOBr/BiOI@ Bi_5_O_7_I was more than 20% higher than that of OBS by BiOI@Bi_5_O_7_I and BiOBr when the OBS concentration was 100 mg/L. In addition, the reaction rate constants of BiOBr/BiOI@ Bi_5_O_7_I were 2.4 and 10.8 times higher than those of BiOI@ Bi_5_O_7_I and BiOBr, respectively. Therefore, the BiOBr/BiOI@ Bi_5_O_7_I ternary heterojunction can be a novel type of heterojunction for the efficient degradation of OBS in water bodies.

## 1. Introduction

**Figure 1:**
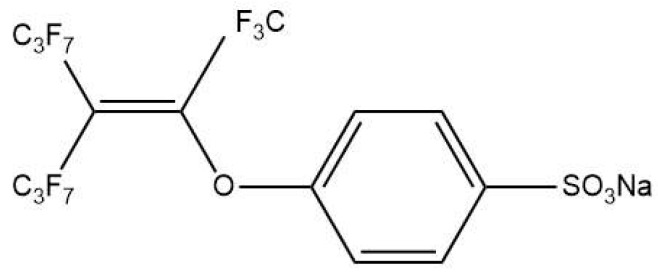
The structure of OBS.

Per- and polyfluoroalkyl substances (PFASs), as linear, branched, or cyclic compounds, have been partially or completely substituted with the F element. PFASs have the most stable C-F (115 kcal/mol) bond and are widely used as surfactants and surface protectants due to their high hydrophobicity, oleophobicity, and thermal and chemical stability [1,2,3]. Sodium p-perfluorous nonenoxybenzenesulfonate (OBS), as an alternative to perfluorooctane sulfonate (PFOS), has been widely used in oil extraction, fire-fighting materials, camera materials, and other industries due to the advantages of easy synthesis and high cost performance and because PFOS has been banned in many countries and regions [4,5,6,7,8]. The structure of the major OBS isomer was shown in Figure 1. The annual output of OBS in China is estimated to be 3500 tons [9].

Thus far, OBS has been found in the Daqing Oilfield, the Shengli Oilfield, the Dagang Oilfield, Poyang Lake, and a wastewater treatment plant in China [4,10,11,12]. Occurrences of OBS have also been found in drinking water, dust, and maternal and umbilical cord serum in China. For instance, 3.2 μg/L of OBS was found in surface water near the Daqing Oilfield, 144 μg/L of OBS was found in wild cross fruits, and 0.711 ng/mL of OBS was even determined in the sera of pregnant women [10,12,13]. However, OBS may cause metabolic disorders in mice, a decrease in hatching and vascular development and metabolic dysfunction in zebrafish, an increase in obesity and a decrease in bone density in humans, and necrosis and oxidative stress in HepG2 cells [4,5,14,15,16,17,18].

Methods for degrading PFOS have been widely studied; however, there are few studies related to the removal of OBS from water bodies [19,20,21,22]. Due to the extra ether group and benzene ring in the structure of OBS, it is more difficult to be degraded naturally compared with PFOS [9,23]. Currently, the removal of OBS from water bodies is mainly through advanced oxidation and adsorption methods. The advanced oxidation method mainly attacks the benzene ring and C=C double bond by generating •OH, SO_4_•^−^, etc., while the adsorption method mainly adsorbs OBS from water through hydrophobic, electrostatic, π-π, and hydrogen bonding interactions [14,15,24,25]. Hwang et al. degraded more than 90% of OBS in 1 h (pH = 11) by adding O_3_ or H_2_O_2_ [14], and Liu et al. removed OBS by adding sulfite under UV irradiation [15]. Li et al. removed 76.6% of OBS in a relatively short period of time by catalytic oxidation using Co@o-MXene combined with peroxymonosulfate (PMS) [24]. In addition, Wang et al. degraded OBS by granular reduced graphene oxide/Fe_3_O_4_ hydrogel, and the OBS removal rate was about 80% in 144 h (pH = 3) [25]. Although advanced oxidation can remove OBS in a relatively short period of time, it often requires the consumption of additional active substances, which increases the cost of degrading OBS. In contrast, the relative cost of removing OBS from water bodies through adsorption is lower, but the reaction time is longer. Therefore, a cost-effective method for the treatment of OBS is warranted.

As a clean and sustainable energy source, the reasonable development of solar energy can solve energy and environmental problems [26]. Photocatalytic degradation technology is considered as a promising technology because of its excellent mineralization ability, extensive energy sources, and mild reaction conditions [27,28,29]. Many photocatalytic studies have been carried on the degradation of PFAS. For instance, Huang et al. prepared an In-MOF/BiOF heterojunction, which completely degraded PFOA (C_0_ = 15 mg/L) within 3 h and PFOS within 1 h under UV light [30]. Park et al. utilized GO/TNA to decompose approximately 82% of PFOA (C_0_ = 15 mg/L) within 4 h of UV irradiation [31]. Zhu et al. adsorbed almost all of hexafluoropropylene oxide-dimer acid (HFPO-DA) (C_0_ = 100 μg/L) within 1 h by the synthesis of Bi/TNTs@AC, and 70.0% of pre-adsorbed HFPO-DA was degraded after 4 h of UV irradiation [32]. Although photocatalytic technology has been applied to the treatment of various PFASs, most studies focused on the UV region, which is not efficient at the removal of pollutants with higher levels. Hence, there is a need for a novel material that might be able to utilize a wider spectral range and perform a higher redox capacity to degrade OBS, as an alternative to PFOS.

Bismuth-based materials are widely used in the fields of water decomposition and pollutant degradation due to their unique stability and economy [33,34,35]. Bismuth halide oxide is a layered structure consisting of a double halogen atom plate with a [Bi_2_O_2_]^2+^ layer, which has enough space to excite the relevant atoms and orbitals and induce the generation of an internal electric field [34]. In particular, BiOI and BiOBr have a narrow band gap and complex energy band structure, which exhibit excellent photocatalytic potential. However, the rapid combination of photogenerated electrons and holes in these materials limits their redox capabilities. To further resolve the said problem, some efficient methods involving constructing heterojunctions, induced defects, crystal surface modulation, and noble metal modification have been widely investigated [34,36].

In general, holes and photogenerated electrons will produce directional motions in response to internal electric field forces by constructing heterojunctions to improve their redox capacity. Long et al. synthesized a BiOBr-Bi_2_S_3_ heterojunction that adsorbed 53.8% of Cr (VI) within 30 min and completely removed it in the subsequent 12 min [37]. Hu et al. degraded 99.2% of rhodamine B within 90 min by synthesizing a Bi_5_O_7_I/BiOBr type-II heterojunction [38]. Due to the synergistic effect between the semiconductors, the ternary heterojunction has a stronger redox capacity compared to the binary heterojunction [39,40]. Zhu et al. synthesized a BiOCl/BiVO_4_/N-GQD ternary heterojunction that degraded more than 70% of bisphenol A in 160 min, which was 1.8 times higher than the reaction kinetic rate constants of BiVO_4_/N-GQD and BiOCl/N-GQD [40]. Shi et al. degraded tetracycline by utilizing a ternary heterojunction of Ag_3_PO_4_/Co_3_(PO_4_)_2_/g-C_3_N_4_, and 88% of tetracycline was removed at 120 min, which was more than 30% higher than that of other heterojunctions [41]. Therefore, the construction of a bismuth oxyhalide-based ternary heterojunction might be considered a promising method of removing contaminants efficiently.

In this study, a BiOBr/BiOI@Bi_5_O_7_I heterojunction was innovatively synthesized and characterized by X-ray diffraction (XRD), X-ray photoelectron spectroscopy (XPS), UV–Vis diffuse reflectance spectroscopy (UV–Vis), etc. The photocatalytic performances were further optimized by the stepwise strategy of single influencing factors for the degradation of OBS, including material, pH, contaminant concentration, and catalyst dosage.

## 2. Materials and Methods

### 2.1. Chemicals and Reagents

Bismuth nitrate hydrate (Bi (NO_3_)_3_•5H_2_O, ≥99.0%, 485 g/mol) and potassium iodide (KI, ≥99.0%, 166 g/mol) were supplied by Damo chemical reagent factory and Technology Development Co., Ltd., (Tianjing, China). Potassium bromide (KBr, ≥99.0%,119 g/mol) was purchased from Sinopharm Chemical Reagent Co., Ltd. (Shanghai, China). OBS was obtained from Shanghai Macklin Biochemical Technology Co., Ltd. (Shanghai, China, ≥95%, 626 g/mol). Ethylene glycol was provided by Tianjin Komeo Chemical Reagent Co., Ltd. (Tianjin, China). All the above reagents were of pure analytical grade. The water used throughout the experiment was ultrapure water.

### 2.2. Synthesis of BiOBr/BiOI@Bi_5_O_7_I

Synthesis of BiOBr (BO): Firstly, 2 mmol of Bi (NO_3_)_3_•5H_2_O was dissolved in 30 mL of an ethylene glycol solution with 2 mmol of KBr. Secondly, after ultrasonication for 20 min, stirring was carried out by a magnetic stirrer, and 2 mmol of the KBr solution was slowly added dropwise into the Bi (NO_3_)_3_ solution. After stirring for 1 h, the above mixed solution was transferred to a 100 mL autoclave and then placed in an oven at a temperature of 160 °C for 12 h. After cooling to room temperature, the obtained material was washed several times with anhydrous ethanol and ultrapure water to obtain the designated BO.

Synthesis of BiOI@Bi_5_O_7_I (BI): Similarly to the BO preparation, KBr was replaced with KI, and then the prepared material was placed in a muffle furnace and heated at 400 °C for 3 h to obtain BI.

Synthesis of BiOBr/BiOI@Bi_5_O_7_I ternary heterojunctions: The above-synthesized BiOI@Bi_5_O_7_I and BiOBr were dispersed in an ethanol solution and sonicated for 1 h. Then, the resulting mixture was transferred to a 100 mL autoclave reactor and heated at 160 °C for 12 h. After cooling down to room temperature, the subsequent mixture was washed with anhydrous ethanol and ultrapure water and dried at 80 °C. The prepared samples were expressed as x BO-(100-x) BI, where x and (100-x) are denoted as the ratios of BiOBr and BiOI, respectively (x = 0, 25, 50, 75, and 100), which could be abbreviated as 25BO-75BI, 50BO-50BI, and 75BO-25BI.

### 2.3. Characterization of the As-Prepared Materials

An X-ray diffractometer with a Cu-Ka emission source was used to examine the 2 θ range of the samples from 5° to 90° to analyze the crystal structure of the materials (Dangdong Tongda Science & Technology Co., Ltd., TD-3500, Dandong, Liaoning, China). Field emission scanning electron microscopy (JEOL Ltd., JSM-IT800, Tokyo, Japan) was used to analyze the microstructure and morphology of the material. The optical properties of the materials were tested by the UV–visible diffuse reflection (Perkin Elmer, LAMBDA 950, Shelton, CT, USA) using BaSO_4_ as a blank reference between 200 and 800 nm. The elemental composition and chemical valence of the photocatalysts of the prepared materials were examined by using X-ray photoelectron spectroscopy (Shimadzu, AXIS SUPRA+, Kyoto, Japan). The specific surface area and particle size distribution of the materials were analyzed with the assistance of the Brunauer–Emmett–Teller method (Micromeritics, ASAP 2460, Norcross, GA, USA).

The band gap energy (E_g_) of the material was deduced from the Tauc equation:

(αhν) ^n/2^ = C (hν − E_g_) (1)

where α, ν, h, and C represent the absorption coefficient, light frequency, Planck’s constant, and the constant value, respectively (direct transition: n = 4, indirect transition: n = 1).

### 2.4. Adsorption Kinetics and Experiments

At 25 °C, 40 mL of a 50 mg/L solution of OBS was placed under dark conditions, and 20 mg of material (BI, BO, 25BO-75BI, 50BO-BI, and 75BO-25BI) was added. It was then mixed with a shaker at 150 rpm, and the change in concentration was measured at a set time.

The equilibrium solid-phase concentration (q_e_) and the reaction rate constants (k_1_, k_2_) were also calculated from the proposed primary and proposed secondary kinetics, respectively.

q_t_ = q_e_ − q_e_exp(−k_1_t) (2)

q_t_ = k_2_q_e_^2^t/(1 + k_2_q_e_t) (3)

where t denotes the time of adsorption and qt is the concentration of OBS in the solid at time t.

### 2.5. Photocatalytic Degradation

The photocatalytic properties of the prepared materials were evaluated under irradiation by a 300 W xenon lamp (China Education Au-light, CEL-PF300-T6, Beijing, China, illumination range 300–1100 nm). All experiments were able to react in a CEL-LB70 photochemical chamber. In this experiment, 20 mg of photocatalyst was added to the OBS solution (40 mL, 50 mg/L). The reaction solution was stirred in the dark for 30 min before exposure to light to bring the reaction to the adsorption–desorption equilibrium. At the set time intervals, the reaction liquid was removed and filtered through a 0.45 μm needle filter. The filtrate was analyzed by a UV spectrophotometer (Shanghai Metash Instruments Co., Ltd., UV-6000PC, Shanghai, China) at 218 nm to determine the concentration change of the OBS.

The kinetics of OBS degradation by the catalyst in the presence of light was determined by examining the concentration of the OBS solution at different times (C) and the concentration of the OBS under the initial conditions (C_0_), as shown in (1):

Photodegradation (%) = C/C_0_ × 100% (4)

The photocatalytic process conforms to the pseudo-first-order kinetic equation, such as the following formula:

−ln (C_t_/C_i_) = K_obs_t (5)

where K_obs_ is the pseudo-first-order rate constant, C_t_ is the OBS concentration at time t, and C_i_ is the OBS concentration at the adsorption equilibrium.

## 3. Results and Discussion

### 3.1. Characterization of Materials

#### 3.1.1. XRD

**Figure 2:**
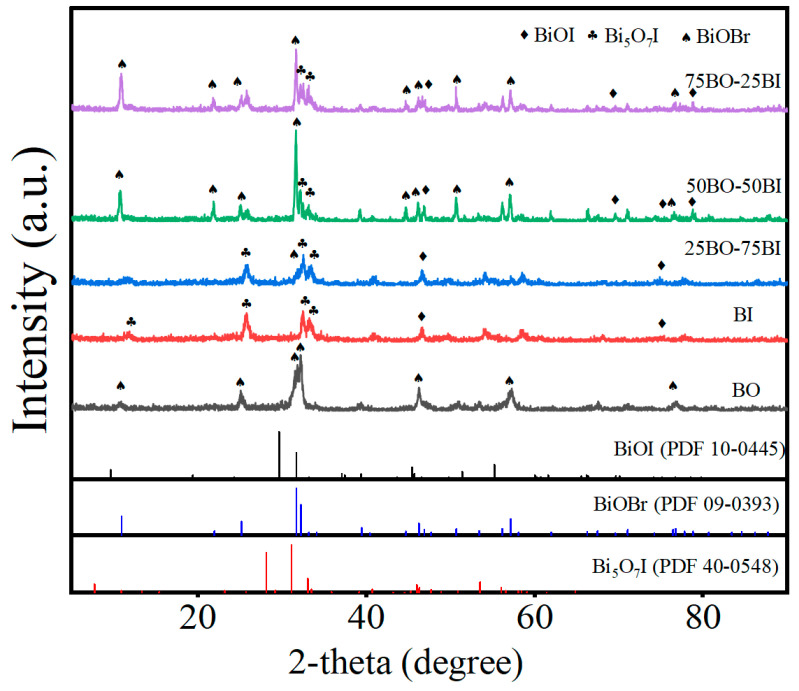
The XRD diagrams of BO, 25BO-75BI, 50BO-50BI, 75BO-25BI, and BI.

The composition and crystal structure of the BO, 25BO-75BI, 50BO-50BI, 75BO-25BI, and BI were investigated by the XRD measurements (Figure 2). The XRD patterns of the synthesized BO were consistent with tetragonal BiOBr (JCPDS 09-0393). The peaks were located at 10.90°, 21.93°, 25.16°, 31.69°, 32.22°, 46.21°, and 57.12°, corresponding to the (001), (002), (101), (102), (110), (200), and (212) facets of the BiOBr. No diffraction peaks of any other phases were observed in the material, demonstrating the effective synthesis of pure BiOBr. In the spectrum of the BI, the peaks at 75.02° and 77.56° may come from the residual BiOI during calcination, and the diffraction peaks were observed at 27.98°, 30.91°, 33.01°, 41.08°, and 46.77°, corresponding to the characteristic peaks of (312), (004), (204), (205), and (604) of Bi_5_O_7_I. In the XRD patterns of the BO, 25BO-75BI, 50BO-50BI, and 75BO-25BI were observed, and the position of the peaks did not change significantly, proving the successful composite of several materials.

#### 3.1.2. SEM

**Figure 3:**
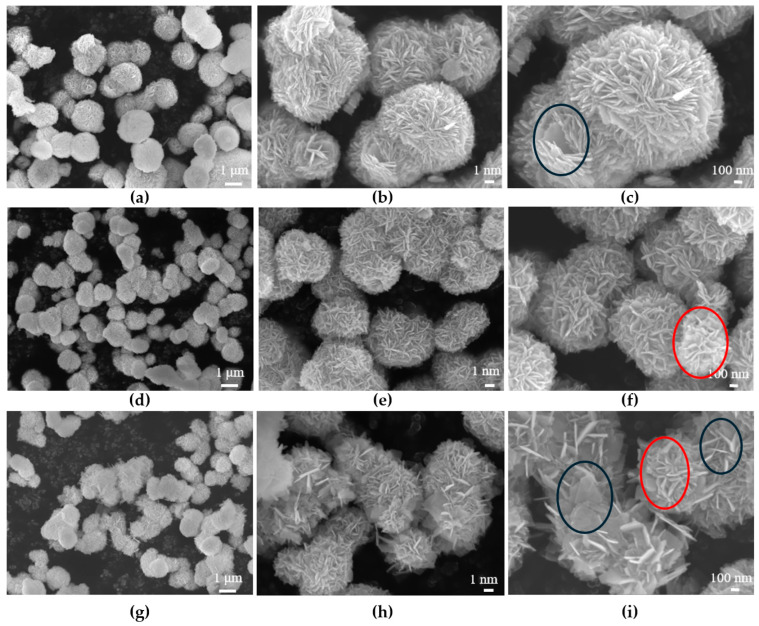
The SEM images of BO (**a**–**c**), BI (**d**–**f**), and 25BO-75BI (**g**–**i**) at different magnifications (The material inside the black and red ovals were BO and BI, respectively).

The morphology and structure of the BO, BI, and 25BO-75BI materials were examined by SEM (Figure 3). It can be observed that several materials are compact microspherical materials around 1–5 μm in volume. The BO is slightly larger than the other two materials, presenting a spherical structure assembled by larger 2D nanosheets (Figure 3a–c). The BI has a smaller and looser structure that contributes to the pore volume and comparative area of the material (Figure 3d–f). As shown in Figure 3i, it is observed that there are two kinds of sheet-shaped structures of both sizes fully contacted to combine into microspheres, which helps the fast transport of charge carriers and also proves the successful combination of both the BI and BO materials.

#### 3.1.3. UV–Vis

**Figure 4:**
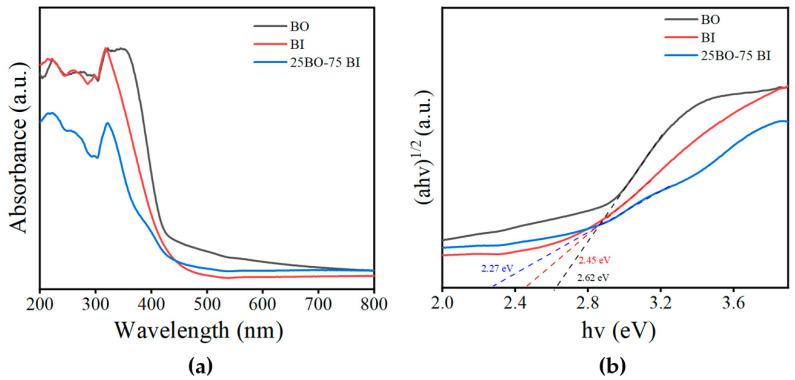
The UV–Vis diffusion reflectance spectra (**a**) and plots of (αhv)^1/2^ vs. the band gap energy (**b**) of the BI, BO, and 25BO-75BI.

The characterization of the optical properties of the BO, BI, and 25BO-75BI was performed by UV–Vis (Figure 4). The difference between the absorption edges of the BO and BI is small. The absorption edge of the BO is 425 nm, while the absorption edge of the BI is 435 nm (Figure 4a). After the two materials were doped with each other, the absorption edge of the 25BO-75BI was slightly red-shifted to about 446 nm, which enhances the absorption of the material in the visible region. In Figure 4b, the band gap energies of the BO, BI, and 25BO-75BI are 2.62 eV, 2.45 eV, and 2.27 eV, respectively. Hence the 25BO-75BI has the narrowest band gap, which can also utilize solar energy more effectively.

#### 3.1.4. XPS

**Figure 5:**
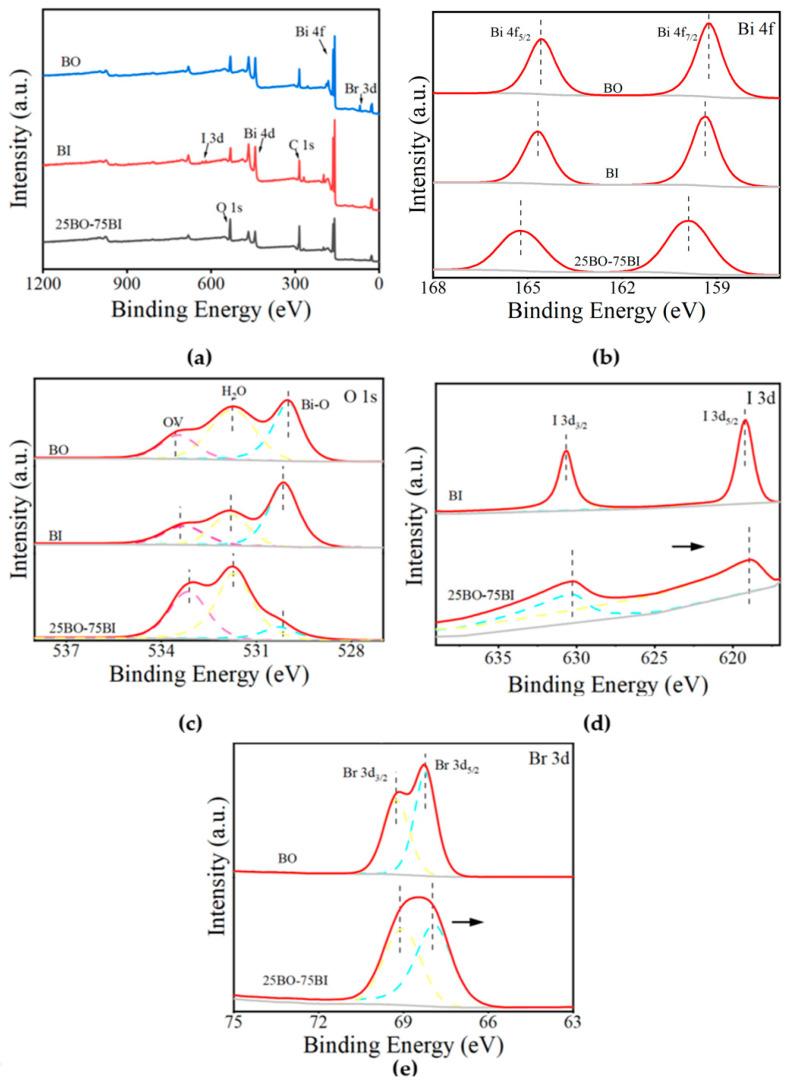
The XPS spectra of BI, BO, and 25BO-75BI: (**a**) full spectrum; (**b**) Bi; (**c**) O; (**d**) I; (**e**) Br (The direction of the black arrow indicated the direction of the change in binding energy, and the dashed lines in different colors represented different peaks of the fit).

In Figure 5 and Table 1, the surface atomic states and elemental valence states of the BO, BI, and 25BO-BI were determined by XPS, and the data were corrected using C 1s (284.80 eV) as a reference peak. The full spectra (Figure 5a) show that the corresponding elements are correctly present in several materials. The 4f_5/2_ and 4f_7/2_ of the BO are located at 159.25 eV and 164.56 eV separately with energy gaps of 5.3 eV, which is consistent with the valence state of Bi (III) [42]. The O 1s signal in the BO is divided into three peaks at 529.99 eV, 531.75 eV, and 533.47 eV, corresponding to the Bi-O bond in the lattice, i.e., the hydroxyl group on the surface of the material, the oxygen atoms around the oxygen vacancies, and the decrease in the Bi-O peak may be due to the absence of O on the surface of the material as a result of warming during the material compounding process [43]. The values 68.24 eV and 69.26 eV in Figure 5d correspond to Br 4f_3/2_ and Br 4f_5/2_, confirming the presence of Br^−^, and the decrease in the peak at 3d_5/2_ of the Br may have produced the electron escape phenomenon [44,45], while the two peaks of BI at 619.21 eV and 630.65 eV correspond to the 3d_5/2_ and 3d_3/2_ orbitals of I, respectively [43,46]. After the two materials were doped with each other, the 4f states of Bi were shifted toward higher binding energy, while I and Br were shifted toward lower binding energy by 0.32 eV and 0.23 eV. This is because I and Br are more capable of trapping electrons compared to [Bi_2_O_2_]^2+^, which leads to a decrease in the charge density and an increase in the binding energy of Bi. It is also demonstrated that the BO and BI are not only physically connected, but a charge transfer occurred between them, proving an interaction between the two materials.

#### 3.1.5. BET

**Figure 6:**
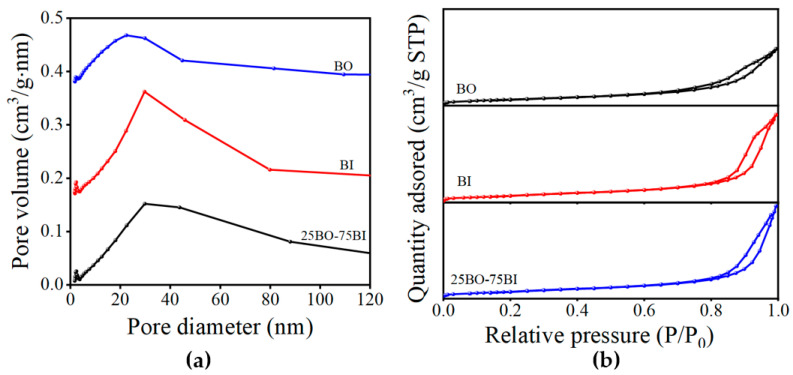
The N_2_ adsorption–desorption isotherm distribution curves (**a**) and pore size distribution curves (**b**) of BI, BO, and 25BO-75BI.

The N_2_ adsorption–desorption isotherms and pore size distribution of the BI, BO, and 25BO-75BI are shown in Figure 6. According to the IUPAC classification, at a given pressure, when the amount of adsorption of all materials increases with increasing equilibrium pressure, the adsorption lines measured do not coincide with the desorption line measured when the pressure decreases. Furthermore, the amount of adsorption in the desorption line is greater than the amount of adsorption at the same relative pressure. Therefore, the adsorption curve of the material conforms to type IV, and the hysteresis loop is type H3 (Figure 6a). The results show that the material is a layered mesoporous structure with a pore size greater than 4 nm and is consistent with the layered structure observed in the SEM. The values of the surface area, pore volume, and pore diameter of several materials are demonstrated in Table 2, where the surface area, pore volume, and pore diameter of the BO are smaller, whereas after doping with BI, the surface area, pore volume, and pore diameter are increased. This provides beneficially sufficient active sites for photocatalytic reactions and adsorption.

### 3.2. Adsorption Kinetics

**Figure 7:**
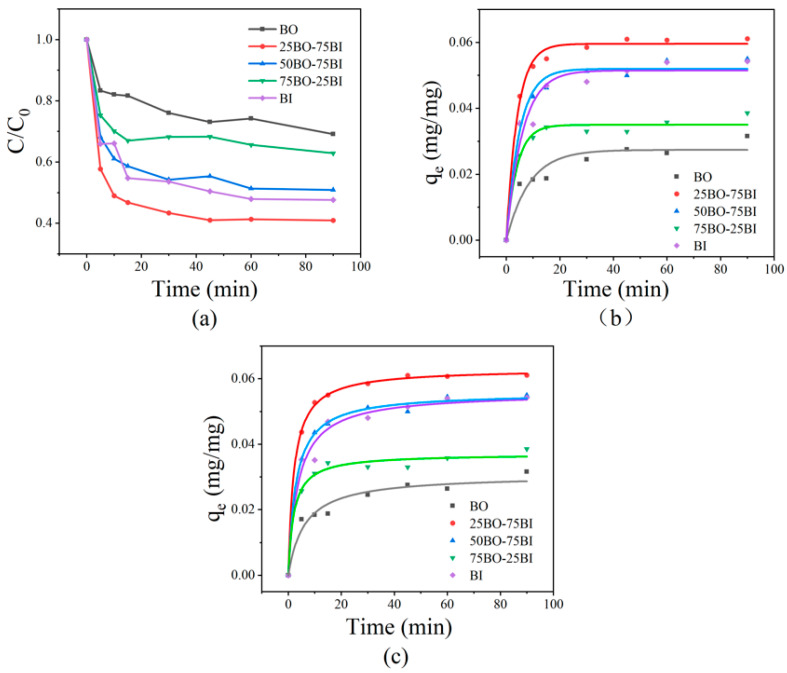
Adsorption curves for different materials (**a**), pseudo-first-order (**b**), and pseudo-second-order kinetics (**c**).

In Figure 7 and Table 3 and Table 4, the OBS adsorption capacity of the BO, 25BO-75BI, 50BO-50BI, 75BO-25BI, and BI are compared according to the adsorption kinetics. The adsorption of OBS by these materials was fast and basically reached the adsorption equilibrium point at 30 min, and the 25BO-75BI and BI had especially better adsorption capacities, which was consistent with the results in BET, in which the 25BO-75BI removed 59.1% of the OBS at 90 min, while the removal of BI reached 52.3% at 90 min.

### 3.3. Photocatalytic Performance

#### 3.3.1. Influence of Material Compositions

**Figure 8:**
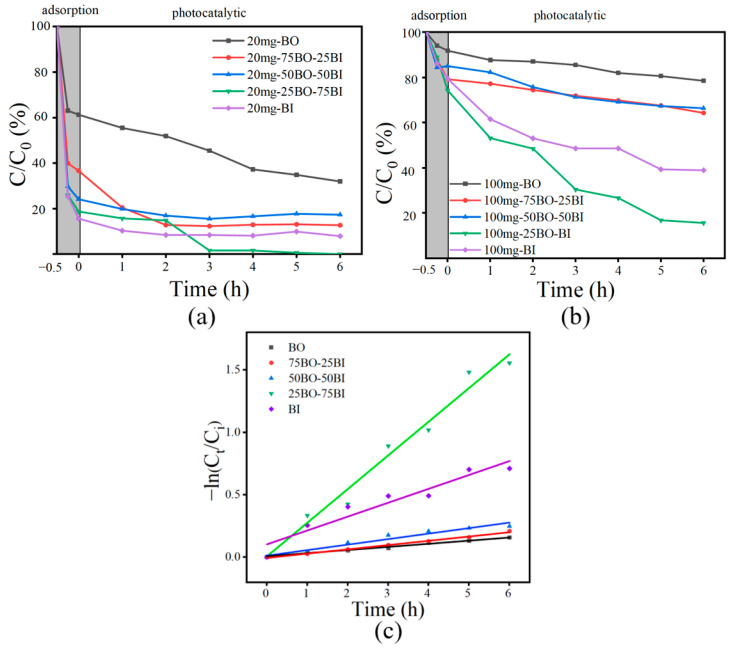
Photocatalytic properties of prepared materials: (**a**) degradation of OBS [OBS = 20 mg/L]; (**b**) degradation of OBS [OBS = 100 mg/L]; (**c**) kinetic curves of different materials [OBS = 100 mg/L].

Comparative studies were implemented on the adsorption degradation effects of BO, 25BO-75BI, 50BO-50BI, 75BO-25BI, and BI at higher and lower concentrations of OBS, together with the effect of BO doping on the adsorption degradation ability of BI (Figure 8). OBS is very stable and hardly degrades in the absence of photocatalysts [17]. As shown in Figure 8, when the concentration of OBS was 20 mg/L, the BO could only remove 38.8% of the OBS in the adsorption stage, while the 25BO-75BI and BI had higher removal rates of 81.3% and 84.3%, respectively. In the photocatalytic stage, the 25BO-75BI was able to remove 99.4% of the OBS at 3 h, which is much better than other types of materials.

In order to further highlight the OBS degradation performance of the different materials, the effects on the treatment of a higher level of OBS (100 mg/L) were compared. When the concentration of OBS was 100 mg/L, the adsorption amount of the 25BO-75BI was close to that of the BI, and the adsorption effect was greater than that of the BO, which might be related to its larger specific surface area. It might provide more adsorption sites, which was consistent with the results of BET. When the concentration of OBS was 100 mg, the removal of OBS by the BO was only 22.4% in 6 h, while the BI could degrade 61.0% of the OBS in 6 h. When the BI was doped with BO at a lower proportion, the removal rate of OBS was significantly increased in the process of lower concentration doping, generating an 84.3% OBS removal in 6 h.

In addition, as shown in Figure 8c and Table 5, the reaction rate constants of the prepared photocatalysts for OBS degradation at 100 mg/L were compared. The highest degradation constant was found for the 25BO-75BI (k_obs_ = 0.26996 h^−1^), which was 2.4 times higher than that of the BI (k_obs_ = 0.11129 h^−1^) and even 10.80 times higher than that of the BO (k_obs_ = 0.025 h^−1^). The results showed that the 25BO-75BI had the highest rate of treating OBS, and a small amount of BO in the BI was effective in improving the photocatalytic effect, which may be related to the synergistic effect between the ternary heterojunction [40]. On the other hand, the removal rate of OBS was not significantly improved by excessive doping, which may be related to the weak redox ability of BO.

#### 3.3.2. Influence of OBS Concentrations

**Figure 9:**
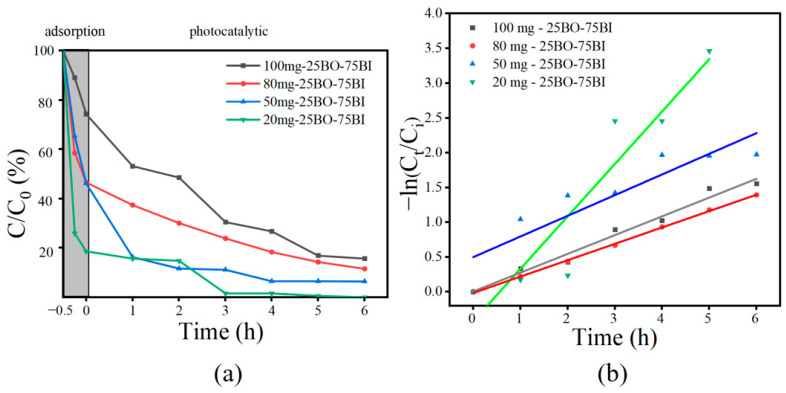
Effect of the initial OBS concentration on OBS degradation (**a**); kinetic curves at different OBS concentrations (**b**).

As shown in Figure 9a, the adsorption degradation effects of the 25BO-75BI at OBS concentrations of 20 mg/L, 50 mg/L, 80 mg/L, and 100 mg/L for 6 h were compared to explore the optimal treatment concentration. In the adsorption stage, the removal rate of OBS could reach 81.3% when the OBS level was 20 mg/L, 53.8% and 53.3% when the OBS level was 50 and 80 mg/L, respectively, and only 26.7% when the concentration of OBS was further increased to 100 mg/L. The results were similar to those of the photocatalytic results. When the concentration of OBS was lower (20 mg/L), the degradation effect of the material was better, and the removal rate reached 100% after 6 h. However, the removal rates could be up to 93.6%, 88.5%, and 84.4% individually when the concentration of OBS gradually increased from 50 mg/L to 100 mg/L. The reason is that the high concentration of OBS will consume the active substances produced by BO in a short time, so that the photocatalytic effect will be reduced [46]. In Figure 9b and Table 6, the fastest reaction rates were observed when the concentrations of OBS were 50 mg/L and 20 mg/L, but the poor fit was mainly due to the fact that the reaction rates decreased significantly when the concentration of OBS was reduced to a certain level. At 20 mg/L of OBS, although the removal effect was excellent, the degradation effect of the material could not be reflected. When the concentration of the contaminant was 50 mg/L, the removal rate was more than 93.6% in 6 h, and the adsorption and degradation ability of the material could be compared, so the concentration of the contaminant was set to 50 mg/L in the subsequent experiments.

#### 3.3.3. Influence of Catalyst Usages

**Figure 10:**
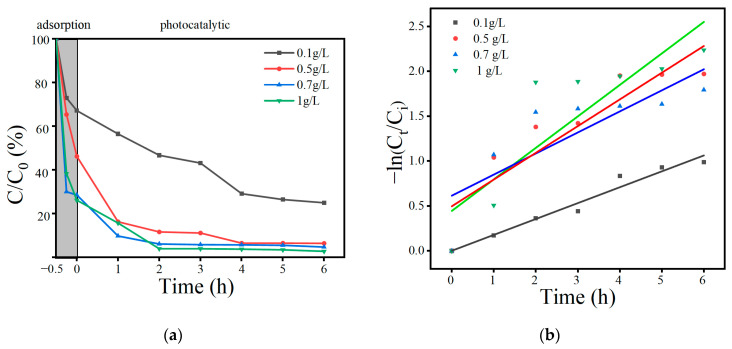
Effect of catalyst usages on OBS degradation (**a**); kinetic curves at different catalyst usages (**b**).

The effects of catalyst dosages of 0.1 g/L, 0.5 g/L, 0.7 g/L, and 1 g/L on photocatalytic degradation were compared in Figure 10a. When the catalyst dosage was 0.1 g/L, only 75.1% of the OBS could be removed in 6 h. With the increase in catalyst dosage, the adsorption effect of OBS was enhanced, and the removal rate of OBS gradually increased from 32.9% to 73.9% within 0.5 h of adsorption. In the photocatalytic stage, when the catalyst dosage was 0.1 g/L, the removal rate of OBS was only 75.1% at 6 h, whereas the removal rates of other dosages could reach more than 95.3%. This may be because the lower catalytic effect of a small amount of catalyst produced fewer free radicals, resulting in a weakened rate. Meanwhile, in Figure 10b and Table 7, when the catalyst was used in the amounts of 0.5 g/L and 1 g/L, it had a faster reaction rate. In order to save resources and ensure the degradation effect, the subsequent catalyst dosage was set at 0.5 g/L. The catalyst dosage was used as a reference for determining the degradation effect. The poorer fits with catalyst amounts of 0.5 g/L, 0.7 g/L, and 1 g/L are also due to the fact that the degradation basically reached the upper limit at the lower concentration of OBS in the first half of the reaction, and the rate became faster in the second half of the reaction.

#### 3.3.4. Influence of Solution pH

**Figure 11:**
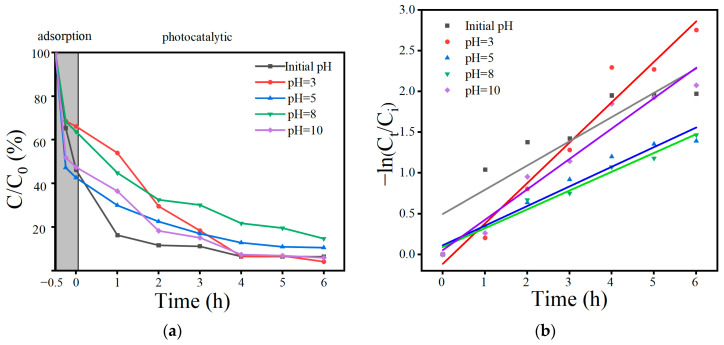
Effect of solution pH on OBS degradation (**a**); kinetic curves at different pH values (**b**).

As shown in Figure 11, the degradation effects of OBS were compared when the solution pH = 3, 5, 8, and 10, and initial pH of 50 mg/L OBS was 6.22. The removal effect was poor in the adsorption stage, with removal rates of 38.8% and 36.8%, respectively, when the solution pH = 3 and 8. However, the removal rates of other materials were higher than 52.5%. In the photocatalytic stage, the fastest removal was achieved at the initial condition, but there was little difference in the removal effect at 6 h. Differences in pH may affect the material electrostatic and π-π effects [25], but it is not obvious in this study. In Figure 11b and Table 8, the fastest reaction rate was observed at a pH = 3. However, there was little difference in the remaining concentration of OBS in the solution when the reaction was carried out for 6 h. Therefore, the pH of the solution was not adjusted in the following experiments.

### 3.4. Photocatalytic OBS Removal Mechanism

**Figure 12:**
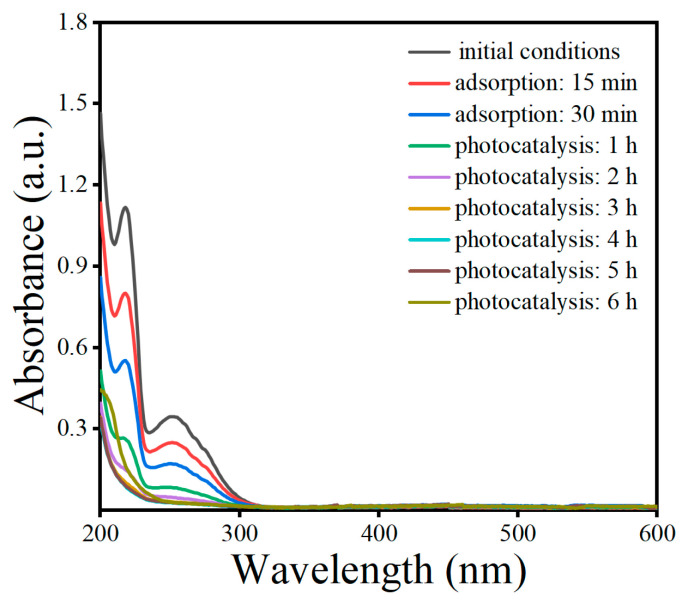
Absorption spectra of OBS solutions at different reaction times.

Under the optimal conditions, a mechanism regarding the removal of OBS was proposed by a UV–Vis spectrophotometric analysis at 200–600 nm (Figure 12). As shown in the figure, the initial OBS solution has two absorption peaks at 218 nm and 254 nm, which correspond to the benzene ring structure and -c=c-, respectively [24,25]. As can be seen from the figure, the wavelength of the OBS at 218 nm gradually decreased in the dark reaction stage, which might be because OBS was adsorbed by the material, resulting in a decrease in the concentration of OBS in the solution. In the photocatalytic stage, the absorption peaks at 218 nm and 254 nm continued to decrease, which might be because the benzene ring and -c=c- were oxidized by the active materials in the photocatalytic process, and the wavelengths were gradually stabilized after 2 h because the reaction had reached the saturation state.

## 4. Conclusions

In the present study, a 25BO-75BI ternary heterojunction was innovatively synthesized, and the photocatalytic ability was enhanced by coupling three semiconductor materials, which could be more economical and efficient for the removal of OBS from aqueous solution. The XRD analysis proved the effective synthesis of the material. The microscopic spherical structure of the material was observed by SEM images. Moreover, the changes in the elemental binding energy of the materials before and after doping were compared through XPS images, indicating a new built-in electric field among the three materials, thus improving the separation of the photogenerated carriers. The UV–Vis test showed that the formation of the ternary heterojunction resulted in a slight increase in the light absorption capacity of the material. The BET test results showed that the 25BO-75BI has a large specific area, which may provide enough active sites for the adsorption and photocatalytic processes. In the adsorption experiments, the solid-phase equilibrium concentration (q_e_ = 0.06307 mg/mg) of the material was determined by adsorption kinetics. In a subsequent degradation experiment, 20 mg/L of OBS was completely degraded by the 25BO-75BI, and the removal rate of OBS at 100 mg/L could reach 84.3%. According to the first-order kinetic analysis, the reaction constants of the 25BO-75BI were 2.4 and 10.8 times higher than those of the BO and BI individually. Finally, the optimal treatment conditions for OBS were explored during this study, showing that 93.6% of OBS was removed at 6 h when the catalyst dosage was 0.5 g/L and the initial concentration of OBS was 50 mg/L.

## Figures and Tables

**Table 1:** Peak splitting results of the XPS spectra.

Element	Elemental Orbital Peaks	BO (eV)	BI (eV)	25BO-75BI (eV)
Bi	4f_5/2_	159.25	159.34	159.86
	4f_7/2_	164.56	164.68	165.18
O	Bi-O	529.99	530.14	530.03
	H_2_O	531.75	531.83	531.79
	OV	533.47	533.29	533.19
I	3d_5/2_	-	619.21	618.98
	3d_3/2_	-	630.65	630.30
Br	4f_3/2_	68.24	-	68.01
	4f_5/2_	69.26	-	69.14

**Table 2:** The N_2_ adsorption characteristics of BI, BO, and 25BO-75BI.

Material	BET Surface Area (m^2^/g)	Pore Volume (cm^3^/g)	Pore Diameter (nm)
BO	23.3838	0.090615	12.3033
BI	26.4047	0.139493	15.8817
25BO-75BI	27.3040	0.148473	17.9120

**Table 3:** Pseudo-first-order reaction rate constants and equilibrium solid-phase concentrations for BO, 25BO-75BI, 50-BO-50BI, 75BO-25BI, and BI determined in 50 mg/L OBS solution.

Material	BO	25BO-75BI	50-BO-50BI	75BO-25BI	BI
K_1_	0.11686	0.24348	0.20256	0.25287	0.16664
q_e_	0.0274	0.5957	0.05195	0.03498	0.05148
R^2^	0.90558	0.99295	0.98327	0.97834	0.95344

**Table 4:** Pseudo-second-order reaction rate constants and equilibrium solid-phase concentrations for BO, 25BO-75BI, 50-BO-50BI, 75BO-25BI, and BI determined in 50 mg/L OBS solution.

Material	BO	25BO-75BI	50-BO-50BI	75BO-25BI	BI
K_2_	6.34445	7.39654	6.18475	13.02043	4.81483
q_e_	0.03063	0.06307	0.05578	0.03705	0.05582
R^2^	0.9521	0.99926	0.99610	0.98332	0.97742

**Table 5:** Rate constants determined of BO, 25BO-75BI, 50-BO-50BI, 75BO-25BI, and BI in 100 mg/L of OBS solution.

Material	BO	75BO-25BI	50-BO-50BI	25BO-75BI	BI
k_obs_ (h^−1^)	0.025	0.03426	0.04409	0.26996	0.11129
R^2^	0.97188	0.99422	0.94666	0.97656	0.92098

**Table 6:** Rate constants at different OBS concentrations.

Concentration	100 mg/L	80 mg/L	50 mg/L	20 mg/L
k_obs_ (h^−1^)	0.26996	0.23552	0.29726	0.75396
R^2^	0.97657	0.99908	0.81387	0.88000

**Table 7:** Rate constants for different catalyst usages.

Catalyst Usage	0.1 g/L	0.5 g/L	0.7 g/L	1 g/L
k_obs_ (h^−1^)	0.17682	0.29765	0.23472	0.35099
R^2^	0.96558	0.81600	0.66118	0.75759

**Table 8:** Rate constants at different pH values.

pH	Initial	3	5	7	10
k_obs_ (h^−1^)	0.29726	0.49649	0.24125	0.37342	0.37342
R^2^	0.81387	0.96358	0.96133	0.95255	0.95255

## Data Availability

The datasets used and/or analysed during the current study are available from the corresponding authors on reasonable request.
